# A combination of monosodium glutamate and high-fat and high-fructose diets increases the risk of kidney injury, gut dysbiosis and host-microbial co-metabolism

**DOI:** 10.1371/journal.pone.0231237

**Published:** 2020-04-08

**Authors:** Thatsanapong Pongking, Ornuma Haonon, Rungtiwa Dangtakot, Sudarat Onsurathum, Apinya Jusakul, Kitti Intuyod, Arunnee Sangka, Sirirat Anutrakulchai, Ubon Cha’on, Somchai Pinlaor, Porntip Pinlaor

**Affiliations:** 1 Biomedical Science Program, Graduate School, Khon Kaen University, Khon Kaen, Thailand; 2 Centre for Research and Development of Medical Diagnostic Laboratories, Faculty of Associated Medical Sciences, Khon Kaen University, Khon Kaen, Thailand; 3 Department of Parasitology, Faculty of Medicine, Khon Kaen University, Khon Kaen, Thailand; 4 Department of Medicine, Faculty of Medicine, Khon Kaen University, Khon Kaen, Thailand; 5 Department of Biochemistry, Faculty of Medicine, Khon Kaen University, Khon Kaen, Thailand; 6 Chronic Kidney Disease Prevention in The Northeast of Thailand, Faculty of Medicine, Khon Kaen University, Khon Kaen, Thailand; 7 Department of Microbiology and Parasitology, Faculty of Medical Science, Naresuan University, Phitsanulok, Thailand; University of Hawai’i at Manoa College of Tropical Agriculture and Human Resources, UNITED STATES

## Abstract

Consumption of either monosodium glutamate (MSG) or high-fat and high-fructose (HFF) diets changes the gut microbiome and hence contributes to development of several diseases. In this study, with an emphasis on kidney injury, hamsters were divided into 4 groups as follows: (1) hamsters fed with standard diet (control); (2) hamsters fed with standard diet and MSG in drinking water (MSG); (3) hamsters fed with high-fat and high-fructose diets (HFF), and (4) animals fed MSG+HFF. After 8 months, the animals were used for the study. Despite showing normal kidney function, hamsters fed with MSG+HFF exhibited signs of kidney damage as demonstrated by the highest expression levels of high-mobility group box-1 and kidney injury molecule-1 in kidney tissues, while slight changes of histopathological features in H&E-stained sections and normal levels of creatinine were observed, indicating possible early stages of kidney injury. Sequencing of the microbial 16S rRNA gene revealed that animals fed with the MSG+HFF diet had a higher ratio of gut Firmicutes/Bacteroidetes along with marked changes in abundance and diversity of gut microbiome compared to hamsters fed with MSG or HFF alone. In addition, ^1^H Nuclear magnetic resonance spectroscopy showed an elevation of urine *p*-cresol sulfate levels in the MSG+HFF group. These results indicate that consumption of both MSG and HFF increases the risk of kidney injury, induces gut dysbiosis and an increase in the amount of *p*-cresol sulfate in hamsters.

## Introduction

Remarkable changes in dietary behaviors, levels of physical activity and prevalence of noncommunicable diseases have been noticed in the world population particularly in low- and middle-income countries [1, 2]. The prevalence of chronic kidney disease (CKD), an important noncommunicable disease, is also increasing worldwide [3, 4]. In Thailand, the overall prevalence of CKD was 17.5% but was higher in Bangkok and the Northern and Northeastern regions of the country [5]. Risk factors including age, gender, diabetes, hypertension, hyperuricemia, history of kidney stones and the use of traditional medicines, are associated with CKD development [5]. Besides these, poor diet is a key risk factor [6]. For instance, consumption of high-fat and high-sugar diets is widely documented as a risk factor for several chronic diseases including CKD [7]. A diet supplemented with high levels of monosodium glutamate (MSG) is also believed to contribute to kidney injury by increasing reactive oxygen species (ROS)-mediated interstitial fibrosis in renal tubules [8, 9], formation of kidney stones [10], and eventually CKD development [11]. Importantly, intake of MSG, along with high-fat and high-fructose diets (HFF), is common in Thailand [12, 13].

The gut microbiota is a complex microbial community comprised of >100 trillion microbial cells. This microbiota plays an important role in human health and disease [14]. The composition of this microbial population can be affected by several factors including antibiotic use, psychological and physical stresses, age, sex, ethnicity, geography, metabolism, immunity and an individual’s diet [15, 16]. Hence, changes in gut microbiota have been considered as important links between diet and chronic disease [6] including CKD [17]. For instance, high consumption of MSG decreases the taxa *Faecalibacterium*, *Megamonas*, *Blautia* in the gut microbiota but leads to an increase of *Collinsella* [18]. Reduction in abundance of *Faecalibacterium* was observed in end-stage CKD patients and was correlated with the reduced estimated glomerular filtration rate in these patients and associated with CKD progression [19]. Long-term consumption of HFF decreases the proportion of Bacteroidetes and increases abundance of both Firmicutes and Proteobacteria in the intestine [20, 21], and eventually leads to increased risk of kidney disease by increasing oxidative stress-induced kidney injury [22, 23]. Besides imbalance of gut microbiota or gut dysbiosis, increases of gut microbiota-derived metabolites including trimethylamine-N-oxide [24], indoxyl sulfate and p-cresol sulfate [25, 26] have been implicated in development and progression of CKD [27].

Although it is well established that the consumption of either MSG or HFF alone affects the gut microbiome and plays a role in kidney disease, it is not known whether consumption of MSG and HFF together enhances severity of kidney injury and gut dysbiosis. In this study, we hypothesized that MSG consumption and an HFF diet, in combination, increase extent of kidney damage and alter the gut microbiome. Accordingly, we aimed to investigate the effect of combined MSG and HFF intake on kidney injury, gut microbiome and gut-derived metabolites in an animal model. This will highlight any risks associated with habitual intake, common in Thailand, of diets high in MSG and HFF, and might be useful in finding strategies for prevention of kidney disease development.

## Materials & Methods

### Ethics statement and experimental design

The protocol of this study was reviewed and approved by the Animal Ethics Committee of Khon Kaen University (IACUC-KKU-22/63) based on the Ethics of Animal Experimentation of National Research Council of Thailand. The animals were obtained from the Animal Unit of Faculty of Medicine, Khon Kaen University. Forty male 6- to 8-week-old Syrian golden hamsters (*Mesocricetus auratus*), average body weight 100–120 g, were housed in eight cages of five animals each. Hamsters were maintained under a standard light cycle (12 h dark/light) and provided with *ad libitum* access to water and food. To avoid bacterial contamination, the stainless-steel cages were washed twice a week with Sunlight detergent (Unilever, Thailand), decontaminated using the antimicrobial reagent Dettol (Dettol, Thailand) and sawdust was changed 3–4 times per week. Hamsters were assigned into four groups, each consisting of 10 animals, and treated as follows for 8 months; 1) normal control group (fed with normal diet); 2) a group fed a high-fat diet along with 10% fructose added in drinking water (HFF); 3) a group given 20 mg/ml of monosodium glutamate in drinking water (MSG), and 4) a group provided with MSG and HFF (MSG+HFF). Hamsters were euthanized at the end of the 8-month experiment.

### Preparation of high-fat diet and monosodium glutamate

The high-fat diet was prepared as previously described [28]. In brief, this diet was prepared by mixing 63.25% of the control diet (Smart Heart, Thailand) with 15% coconut oil, 15% corn oil, 0.5% cholesterol (Sigma-Aldrich, St. Louis, MO, USA), and 0.25% deoxycholate (Sigma-Aldrich), 6% tapioca starch (Fish Band, Thailand). The fat composition of this diet was analyzed by the Central Laboratory, Co. Ltd. (Bangkok, Thailand) (https://www.centrallabthai.com/index.php/th/) using AOAC official methods of analysis. The 10% fructose solution in drinking water was prepared using purified fructose (Merck Millipore, Darmstadt, Germany). Monosodium glutamate was prepared by adding 20 mg/ml of MSG into drinking water (commercial grade, Bangkok, Thailand).

### Sample collection and DNA extraction

To avoid bacterial contamination, feces were collected directly from the rectum at the end of the experiment and kept at -20 °C until analysis. DNA was extracted from feces using the TIANamp Stool DNA kit (Tiangen Biotech, Beijing, China) according to the manufacturer’s instructions. The concentration of extracted DNA was measured using a Nanodrop2000 spectrophotometer (NanoDrop Technologies, Wilmington, DE, USA). DNA samples of equal amounts from individual hamsters were pooled by experimental group and kept at -20 °C until analysis. Kidney tissue was kept in 10% formalin, followed by processing into paraffin blocks for immunohistochemistry analysis.

### Biochemical measurement

The creatinine (Cr) level in serum, a marker commonly used for evaluation of renal function, was measured using enzymatic and colorimetric methods (Cobas 8000 Modular Analysis Series, Roche Diagnostics, Bangkok, Thailand). The data were reported as means ± SD. To compare among different experimental conditions, data were analyzed using analysis of variance (one-way ANOVA) with post-hoc Tukey’s HSD (Honestly Significant Difference) test. The analyses were performed using GraphPad Prism version 8.02 (GraphPad Software, La Jolla, CA, USA).

### 16S rRNA gene sequencing and analysis

A hypervariable region of prokaryotic 16S rRNA (V3-V4 regions) was amplified from fecal DNA by PCR as described previously [29] using a thermal cycler and an Expand high-fidelity PCR system (BioRad C1000TM Thermal Cycler). A quantity of PCR product was electrophoresed in a 1.5% agarose gel to confirm the size of the product, expected to be about 450–500 bp.

The partial 16S rRNA gene was sequenced using Hiseq2500 (Illumina Inc., California, USA). The 16S sequencing library was prepared by fragmentation of genomic DNA (gDNA) and ligating with specialized adapters to both fragment ends. Paired-end reads were assigned to samples based on their unique barcode and truncated by cutting off the barcode and primer sequences. Paired-end reads which partially overlapped were merged using FLASH (V1.2.7, http://ccb.jhu.edu/software/FLASH/) [30] to produce raw tags. Quality filtering on the raw tags was performed under specific filtering conditions to obtain high-quality clean tags [31] according to the Qiime (V1.7.0, http://qiime.org/scripts/split_libraries_fastq.html) quality-control process. The tags were compared with the reference database (Gold database, http://drive5.com/uchime/uchime_download.html) using the UCHIME algorithm (UCHIME Algorithm, http://www.drive5.com/usearch/manual/uchime_algo.html) [32] to detect chimeric sequences, which were then removed [33]. All tags that successfully passed through these filtering processes were then analyzed using Uparse software (Uparse v7.0.1001 http://drive5.com/uparse/) [34]. Sequences sharing ≥97% similarity were assigned to the same OTU. A representative sequence for each OTU was analyzed for further annotation. Each representative sequence was screened against the SSUrRNA database of the SILVA database (http://www.arb-silva.de/) [35] using Mothur software for annotation at each taxonomic rank (Threshold:0.8~1) [36] (kingdom, phylum, class, order, family and genus). Information on abundance of each OTU was normalized relative to the sample with the fewest sequences. Subsequent analyses of alpha and beta diversity were all performed basing on this normalized data. Alpha diversity indicates species diversity for a sample through two indices, including observed-species and Shannon. We calculated these indices using QIIME (Version 1.7.0) [37] and displayed results using R software (Version 2.15.3) [38]. Beta diversity analysis was used to evaluate differences between samples in species complexity: weighted UniFrac values were calculated using QIIME software (Version 1.7.0) [37].

### Sample preparation for NMR analysis

Urine was drawn directly from the urinary bladder using a sterile syringe at the end time point of experiment and kept at -80 °C. Samples from each hamster were thawed at room temperature and spun at 18,000 g at 4 °C for 10 min. One hundred and eighty μL of the supernatant was thoroughly mixed with 20 μL of 1.5 M potassium phosphate buffer (pH 7.4) containing 100% D_2_O, 0.13 mg/mL NaN_3_, 1 mg/mL of TSP (3-(trimethylsilyl) propionic-2,2,3,3-d4 acid sodium salt). Finally, 180 μL of the mixture was transferred into a 3 mm NMR tube using an automatic syringe (eVol^®^) and placed in SampleJet racks (4°C) until the NMR spectroscopic measurements.

### ^1^H NMR data acquisition and statistical analysis

Urine samples were analyzed using a Bruker AVANCE III 600 MHz 1H NMR spectrometer (Bruker; Rheinstetten, Germany), operating at 600.13 MHz at a temperature of 300° K using a standard NMR pulse sequence (relaxation delay-90°-t-90°-t_m_ (mixing time)-90°-acquisition) to acquire one-dimensional spectral data. The parameters used are described elsewhere [39].

^1^H NMR spectral data were automatically pre-processed (phasing, baseline correction and calibration to TSP) in Topspin 3.6.0. The spectral data were then imported into MATLAB (version R2018a) for statistical analysis. The regions containing water (δ1H 4.70–4.90), urea (δ1H 5.48–6.28) and TSP (δ1H -0.20–0.20) were removed. Alignment was applied using recursive segment-wise peak alignment method [40] and aligned data were normalized using the probabilistic quotient method prior to multivariate data analyses. Principal component analysis (PCA) and orthogonal projection to latent structures−discriminant analysis (OPLS-DA) were applied to compare ^1^H NMR spectral data between the different groups. Metabolites that contributed to group discrimination were identified based on a previously published study [41, 42] and confirmed with STOCSY, Chenomx NMR suite 8.3 software (Chenomx Inc. Edmonton, Alberta, Canada) and The Human Metabolome Database (HMDB, http://www.hmdb.ca/).

### Histological study

Kidney tissues were fixed in formalin and paraffin-embedded sections were cut at 4 μm thickness, deparaffinized in xylene and rehydrated using a series of graded ethanols (absolute ethanol, 95% alcohol and 70% alcohol) to distilled water. Subsequently, slides were stained with hematoxylin for 10 min and washed in running tap water for 2 min. Slides were destained in acidified alcohol (1% acid in 70% ethanol), rinsed with running tap water and blued in saturated lithium carbonate for 3–4 sec. Then, the slides were rinsed again for 10–20 min before staining with eosin for 15–20 sec, dehydrated and mounted.

### Immunohistochemistry

Kidneys from six individuals per experimental group were subjected to immunohistochemistry. Kidney sections (4-μm thickness) were deparaffinized, rehydrated and then antigens retrieved by autoclaving in citrate buffer pH 6.0 for 15 min. Subsequently, endogenous peroxidase activity was blocked by immersing the slides in a solution of 3% hydrogen peroxide in methanol. After blocking with 5% fetal bovine serum (FBS), the sections were incubated with a 1:20 dilution of mouse monoclonal kidney injury molecule-1 (KIM-1) antibody (R&D Systems, Abingdon, UK) and a 1:350 dilution of rabbit polyclonal anti-high mobility group box-1 (HMGB-1) antibody (Abcam, Cambridge, UK) in 1% FBS overnight in a humidified chamber at 4 °C. After washing with phosphate buffer saline solution, slides were incubated with a 1:400 dilution of HRP-conjugated sheep anti-mouse IgG (Jackson ImmunoResearch, West Grove, PA, USA) and 1:1000 dilution of HRP-labeled goat anti-rabbit IgG (Jackson ImmunoResearch, West Grove, PA, USA) for 1 h at room temperature. The immunoreactivity signal was generated using diaminobenzidine substrate and counterstained with Mayer’s hematoxylin. Localization of immunohistochemical staining was assessed using light microscopy. Brown staining in the cytoplasm of cortical tubules was regarded as indicating the presence of KIM-1. HMGB1 was observed in the nucleus of vascular, proximal and distal tubular cells. Ten fields (200× magnification) of slides stained for both KIM-1 and HMGB-1 were randomly selected for analysis by ImageJ software (National Institutes of Health, Bethesda, MD, USA) [43].

### Statistical analysis

The scores for percentage-positive areas of both KIM-1 and HMGB-1 tissues from individual hamsters met the conditions for normality according to a Shapiro-Wilk test. Means of these scores were therefore compared between experimental groups using one-way ANOVA (Analysis of Variance) with a post-hoc Tukey HSD (Honestly Significant Difference) test. A *P*-value of less than 0.05 was considered to indicate a significant difference between groups. All analyses were performed using SPSS version 26.0 (IBM, Armonk, NY, USA) and GraphPad Prism version 8.02 (GraphPad Software, La Jolla, CA, USA).

## Results

### A combination of MSG and HFF diets exacerbates kidney pathology without affecting serum creatinine levels

**Fig 1:**
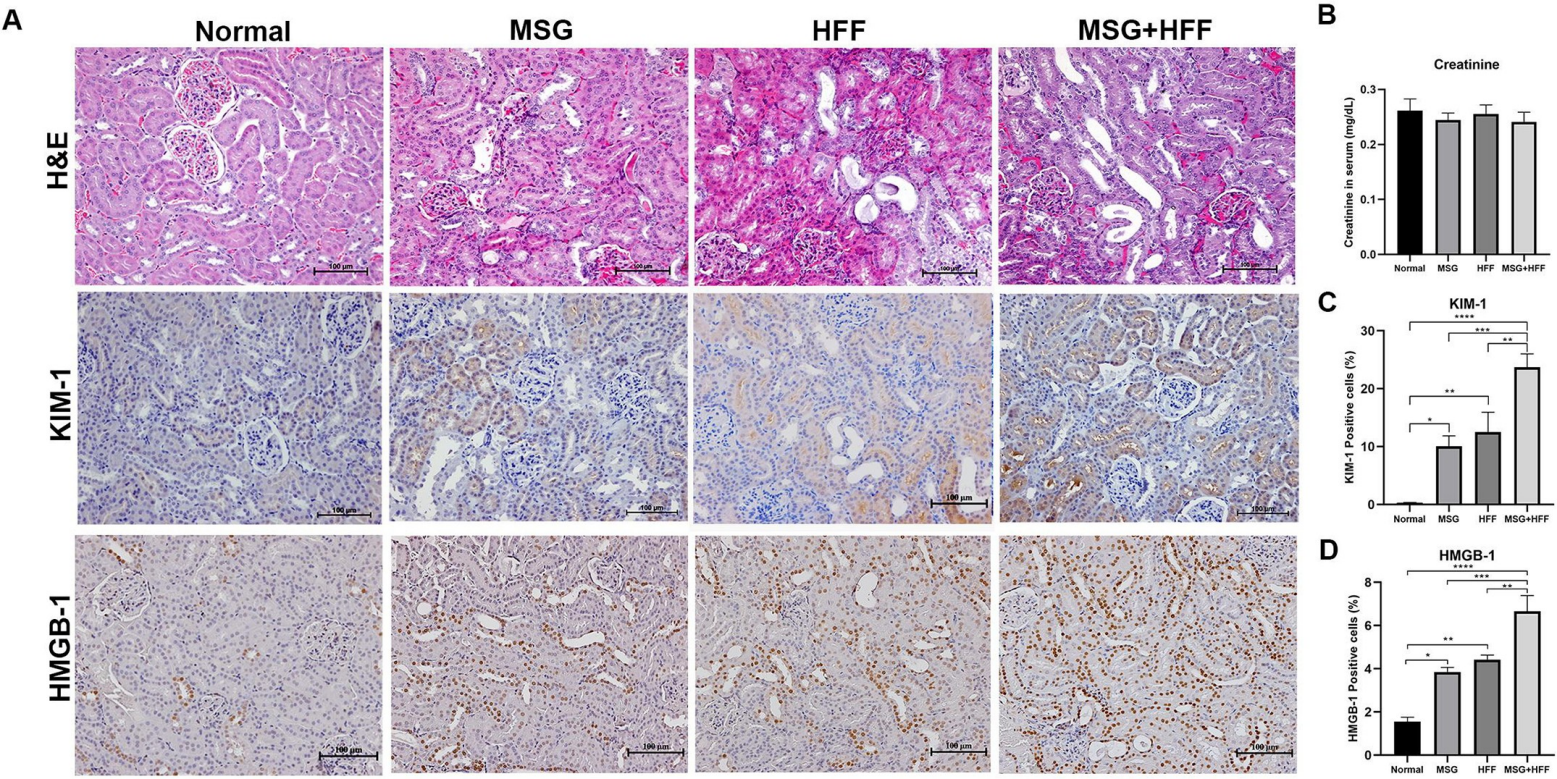
(A) Representative photographs of sections of hamster kidneys stained by H&E and for expression of kidney injury molecule-1 (KIM-1) and high-mobility group box 1 protein (HMGB1), (B) serum creatinine levels and percentages of (C) KIM-1- and (D) HMGB1-positive cells. The extent of injury in these kidneys was evaluated after 8 months on experimental diets including the normal group (n = 6), monosodium glutamate-treated group (MSG, n = 6), high-fat and high-fructose diet-treated group (HFF, n = 6) and the MSG and HFF diet-treated group (MSG+HFF, n = 6). Findings are presented as mean ± SEM. *, **, *** are *p* < 0.05, *p* < 0.01 and *p <* 0.0001, respectively, compared with normal control or MSG or HFF.

In the histological study by H&E (Fig 1A), the normal control hamsters showed normal tubular cells and glomeruli, while mild, moderate and severe renal tubular dilatation were found in hamsters treated with MSG, HFF and MGS+HFF, respectively. Next, we sought to evaluate renal function by measurement of creatinine level in serum. This level, in all experimental groups, was within the reference range for hamsters (0.4–1.0 mg/dL) [44] and did not statistically differ among groups (*p* > 0.05) as shown in Fig 1B.

### A combination of MSG and HFF diets increases KIM-1 and HMGB-1 expression in renal tissue

Base on histopathological demonstration on stained slides, we evaluated location and expression levels of KIM-1 and HMGB-1, renal injury markers, using immunohistochemistry (Fig 1). Expression of KIM-1 was observed mainly in the cortex on the apical side of proximal tubules. KIM-1-positive cells were observed in membranes and cytoplasm of proximal tubular cells with some regional differences within tubules. In comparison to normal control hamsters, the percentage of the positive area of KIM-1 was statistically significantly higher in all groups fed with a modified diet: MSG (*p <* 0.05), HFF (*p* < 0.005) and MSG+HFF (*p* < 0.0001) (Fig 1C). However, the expression levels of KIM-1 and HMGB-1 between the MSG and HFF groups were not statistically different (Fig 1C and 1D). Expression of both marker molecules was significantly higher in the MSG+HFF group than in all other groups (Fig 1C and 1D).

### A combination diet of MSG and HFF changes the gut microbiota composition

**Fig 2:**
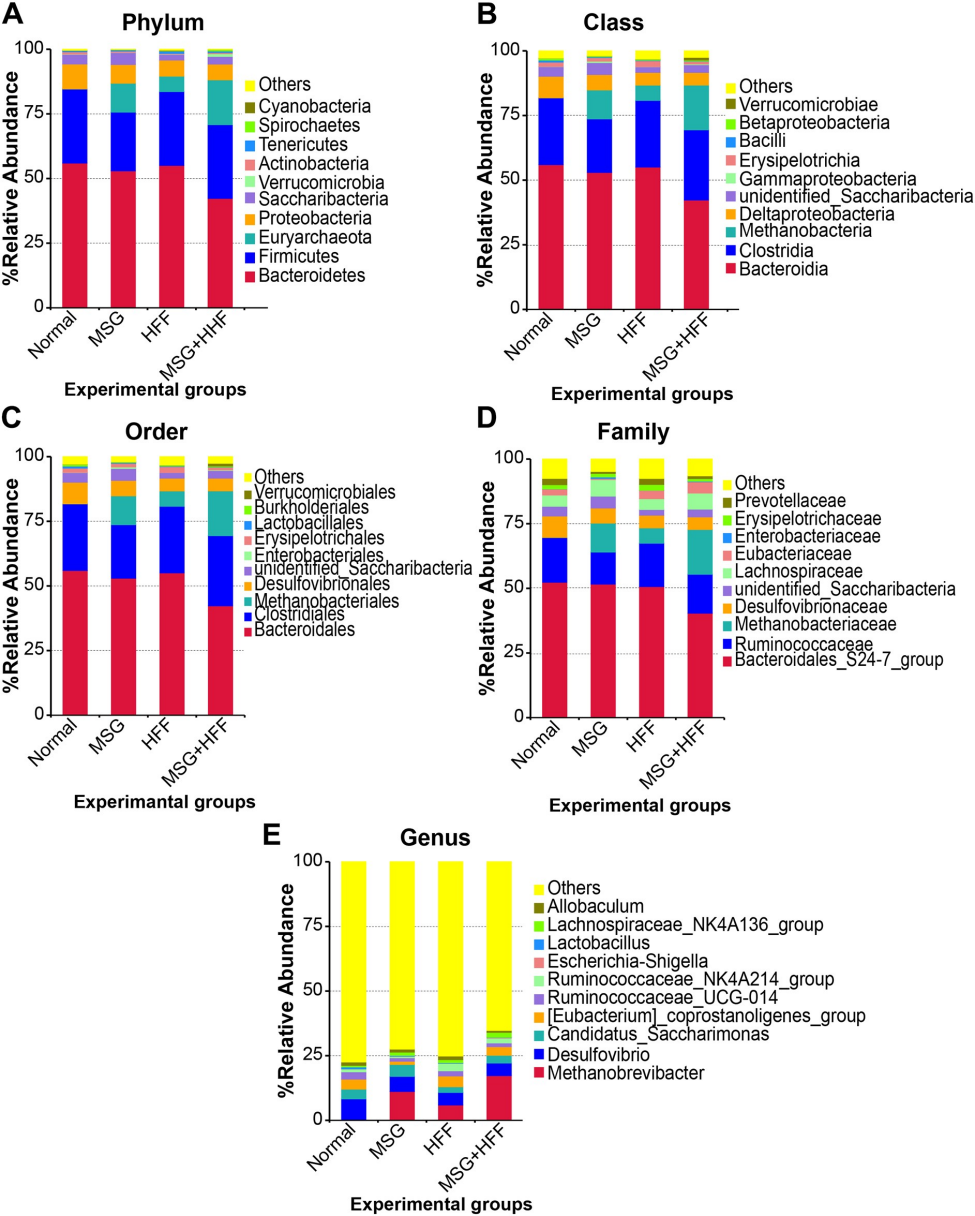
Fecal microbial composition in hamsters at different taxonomic levels. The columns represent a normal group (n = 10), monosodium glutamate-treated group (MSG) (n = 10), high-fat and high-fructose diet-treated (HFF) (n = 10) and MSG and HFF diet-treated (MSG+HFF) (n = 10).

Fifteen microbial phyla, 28 classes, 39 orders, 65 families and 150 genera were detected across all samples. To show the relative abundance of bacterial communities more intuitively, we selected the top 10 species identified in each sample or group and generated percentage stacked histograms of relative abundance at phylum, class, order, family and genus levels (Fig 2).

At the phylum level (Fig 2A), Bacteroidetes, Firmicutes and Proteobacteria were abundant in all experimental groups. The abundance of Euryarchaeota was higher in groups fed modified diets; 5.94% for HFF, 11.17% for MSG, and 17.32% for the MSG+HFF group, while this phylum constituted only 0.05% of reads in the normal control group. Notably, Bacteroidetes slightly decreased from 55.99% in the normal control to 55.06% in HFF, 52.98% in the MSG group and to 42.32% in the MSG+HFF group. Likewise, the abundance of Firmicutes decreased from 28.63% in the normal control group to 22.71% in the MSG group. The ratio of Firmicutes/Bacteroidetes was higher in the MSG+HFF group (0.675) compared to either MSG (0.429), HFF (0.519) or normal control (0.511) groups.

At the class level (Fig 2B), the relative abundance of the top 4 bacterial classes was observed in the order Bacteroidia > Clostridia > Deltaproteobacteria > Unidentified_Saccharibacteria in all experimental groups. Methanobacteria was observed only in groups fed the modified diets. The relative abundance of Bacteroidia showed a decreasing trend from 55.99% in the normal control group to 55.06% in HFF, 52.98% in MSG and 42.32% in the MSG+HFF groups. On the other hand, the proportion of Methanobacteria increased from 0.05% in the normal control group to 5.92% in HFF, 11.17% in MSG and 17.32% in the MSG+HFF group. Notably, abundance of reads from Methanobacteria in the MSG+HFF group was 346.40-fold higher than in the normal control group, 118.4-fold higher than in the MSG group and 223.4-fold more than in the HFF group.

At the order level (Fig 2C), the most abundant taxa were observed in the following order in all experimental groups: Bacteroidales > Clostridiales > Desulfovibrionales > Unidentified_Saccharibacteria. Methanobacteriales was observed only in groups fed a modified diet. Notably, in the MSG+HFF groups, Bacteroidia was least abundant and Methanobacteria was most abundant. Abundance of Desulfovibrionales decreased from 8.32% in the normal control group to 5.96% in MSG, 4.92% in HFF and 4.89% in the MSG+HFF group.

The four most abundant families (Fig 2D) were observed in all experimental groups in the following order: Bacteroidales_S24-7_group > Ruminococcaceae > Desulfovibrionaceae > Lachnospiraceae. The MSG+HFF group had the lowest level of Bacteroidales_S24-7_group, Desulfovibrionaceae and Erysipelotrichaceae, but the highest abundance of Eubacteriaceae and Methanobacteriaceae.

As shown in Fig 2E, the top four gut bacterial genera found in all experimental groups were *Desulfovibrio* (MN326533), *Ruminococcaceae*_UCG-014 (MN326541), and the [*Eubacterium*]_coprostanoligenes_group (MN326531). In the MSG+HFF group, *Methanobrevibacter* (MN326530), *Lachnospiraceae*_NK4A136_group (MN326551) and *Escherichia-Shigella* (MN326536) were more abundant than in the other groups, while *Lactobacillus* (MN326537), *Desulfovibrio* (MN326533), *Ruminococcaceae*_UCG-014 (MN326541) and *Allobaculum* (MN326542) were less abundant.

**Fig 3:**
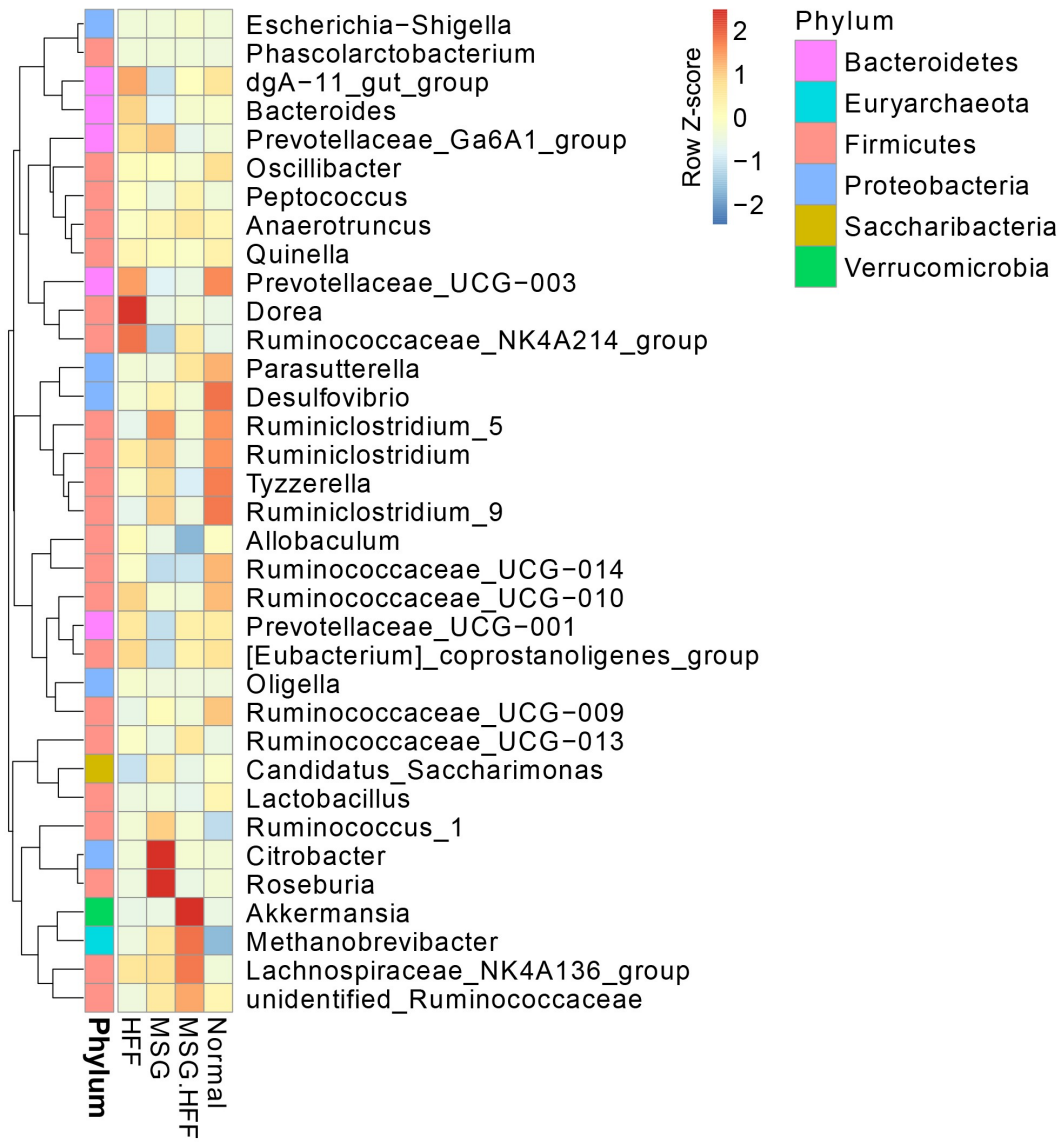
Composition of the gut microbiota. Heat map of the top 35 bacterial genera identified from hamster fecal DNA. The abundances were arranged using unsupervised hierarchical cluster analysis (blue, low abundance; red, high abundance).

A heatmap of the top 35 genera observed in all groups is shown in Fig 3. Relative to the normal control group, *Citrobacter* (MN326558), *Roseburia* (MN326554) and *Ruminococcus*_1 (MN326532) were found to have the highest level in the MSG treated group. Similarly, *Citrobacter* (MN326558) was 58-fold more abundant in the MSG group. In the HFF group, *Dorea* (MN326559), *Ruminococcaceae*_NK4A214_group (MN326535), dgA-11_gut_group (MN326549), *Desulfovibrio* (MN326533), *Bacteroides* (MN326550) and *Oligella* (MN326546) were present in greatly increased abundance. In addition, *Methanobrevibacter* (MN326530), *Akkermansia* (MN326539), *Escherichia-Shigella* (MN326536), *Lachnospiraceae*_NK4A136_group (MN326551), unidentified*_Ruminococcaceae* (MN326547), *Anaerotruncus* (MN326560), *Peptococcus* (MN326556) were highly elevated (relative to the normal control group) in the MSG+HFF group by 372.54, 13.15, 3.42, 3.19, 1.40, 1.15 and 1.81-fold, respectively. In contrast, *Lactobacillus* (MN326537), *Tyzzerella* (MN326538), *Ruminiclostridium* (MN326563), *Allobaculum* (MN326542), *Prevotellaceae*_Ga6A1_group (MN326553) and *Quinella* (MN326546) were less abundant by 6.74, 3.76, 2.17, 1.75, 1.33 and 1.28-fold, respectively, in the MSG+HFF group when compared to the normal group. Interestingly, in the MSG+HFF group, the genera *Akkermansia* (MN326539) and *Escherichia-Shigella* (MN326536) were 14.10- and 3.90-fold, respectively, more abundant than in the MSG group and 15.18- and 8.70-fold, respectively, more abundant than in the HFF group.

**Fig 4:**
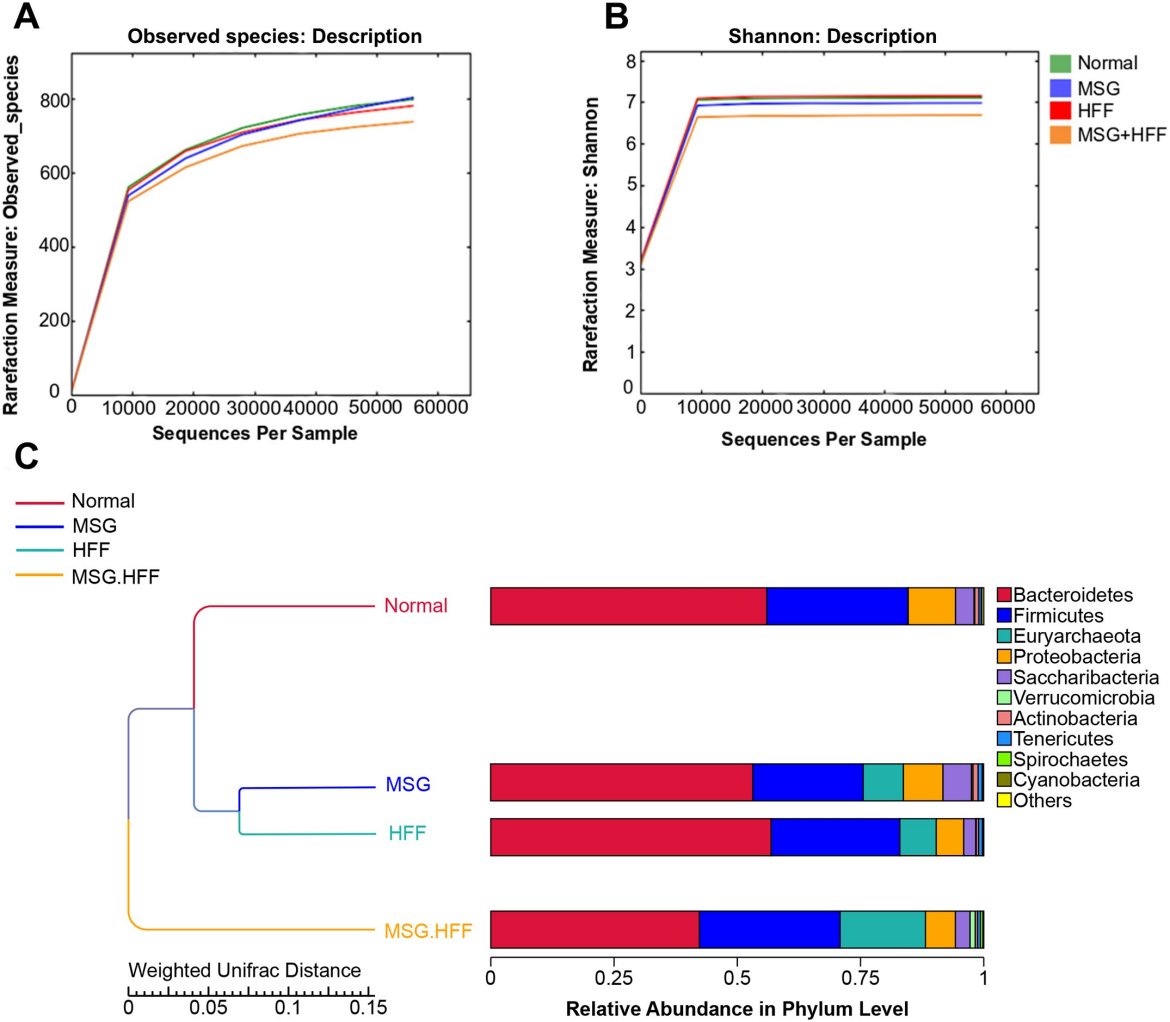
Alpha diversity of the sequence reads from DNA extracted from hamster feces for each group. (A) rarefaction analysis of the observed taxa and (B) the Shannon index. (C) Beta diversity, UPMGA clustering trees-weighted Unifrac distance. The results of clustering using two distance matrixes were combined with the overall percentages of relative abundance among all samples at phylum level.

Fig 4 shows alpha and beta gut microbial diversity. Alpha diversity did not differ among the normal, MSG and HFF groups but was lower in the MSG+HFF group (Fig 4A and 4B). The weighted UniFrac distance cluster analysis (UPGMA) was used to show the similarity between the different experimental groups. The bacterial communities were more similar in the normal control, MSG and HFF groups relative to the MGS+HFF group (Fig 4C).

### A combination of MSG and HFF diets changes urine metabolite levels

^1^H NMR spectroscopy was used to obtain urine metabolite profiles from each group of hamsters (S1 Fig). Three metabolites, trimethylamine *N*-oxide (TMAO), indoxyl sulfate and *p*-cresol sulfate involved in metabolite-related kidney injury, were selected for presentation in Table 1.

**Table 1:** Pairwise comparisons of differentially produced urine metabolites among experimental groups.

Metabolites	N/MSG	N/HFF	N/MSG+HFF	MSG/HFF	MSG/MSG+HFF	HFF/MSG+HFF
TMAO	↑	↑	↓***	↓*	↓*	↓
Indoxyl sulfate	↑	↓***	↓**	↓***	↓**	↑
*p*-cresol sulfate	↓	↑***	↑*	↑***	↑*	↓*

Arrows represent any metabolic changes, associated with specific diets in hamster urine in pairwise comparisons among experimental groups. Significant differences are indicated by *, **, and *** which correspond to *p* < 0.05, < 0.01, and < 0.001, respectively. TMAO; trimethylamine *N*-oxide, N; Normal; MSG, monosodium glutamate-treated group; HFF, high-fat and high-fructose diet-treated; MSG+HFF, group receiving a diet containing both MSG and HFF.

The level of TMAO was significantly lower in urine from the MSG+HFF group (*p* < 0.05) when compared to the normal control and MSG groups. Levels of indoxyl sulfate were also significantly lower in the HFF group (*p <* 0.001) and the MSG+HFF group (*p <* 0.01) compared to the normal control and MSG groups. In contrast, compared to normal controls, the level of *p*-cresol sulfate was significantly higher in the HFF group (*p* < 0.001) and in the MSG+HFF group (*p* < 0.05), but was lower in the MSG group. Likewise, levels of this urine metabolite were significantly higher in the HFF group (*p* < 0.001) and the MSG+HFF group (*p* < 0.05) when compared with the MSG group alone.

## Discussion

**Fig 5:**
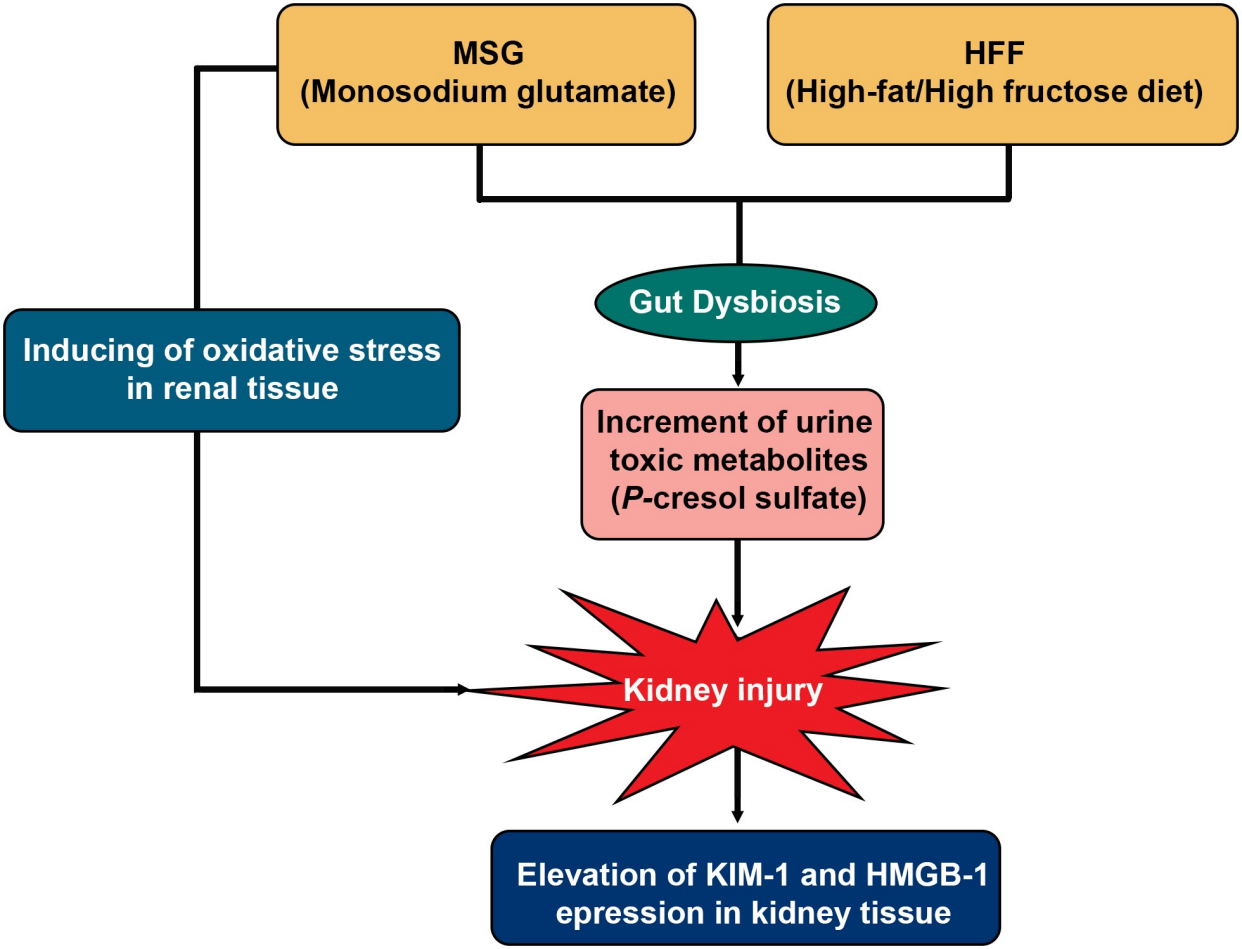
Postulated mechanism by which the MSG+HFF diet might cause kidney injury.

Alteration of gut microbiota composition results in a change in gut-derived metabolites, some of which are uremic toxins that can induce renal damage [45, 46]. Here, we have reported the effects of MSG in combination with HFF diets on kidney injury and co-observation of gut dysbiosis and urine metabolite alteration in hamsters. The MSG dose (20 mg/ml) was used based on an average MSG intake in humans (0.4–14 gm/day) [12]. A combination of MSG and HFF diets led to greater damage to kidney tissues, alteration of the gut microbiota, decrease in urine trimethylamine *N*- oxide (TMAO) and indoxyl sulfate and increase of *p*-cresol sulfate production relative to animals fed MSG or HFF alone. Increased levels of blood uremic toxins such as *p*-cresol sulfate might cause kidney damage in our animal model as reflected by the greater amount of this metabolite in urine. A postulated mechanism of kidney injury is summarized in Fig 5.

It is well known that consumption of MSG or a HFF diet has the potential to induce kidney injury, reflected in changing histology in kidney tissues [47–49]; however, there is no information on the effect of these in combination on kidney pathology. Here we present such information for the first time. We found that an MSG+HFF diet exacerbated kidney injury, such as tubular dilatation, more than did a single diet type. One unexpected result was that serum creatinine did not deviate from normal controls, suggesting that any injury to kidney tissue in this model must be at an early stage. That some injury occurring was supported by the high expression levels of the renal injury markers, KIM-1 and HMGB-1 [50, 51]. These markers might prove useful for diagnosis of early kidney damage.

Many reviews have revealed that alteration of gut microbiota plays an important role in causing kidney injury [14, 27, 52]. Hence, we further verified the effect of this change in our animal model. Sequencing of prokaryotic 16S rRNA sequences (V3-V4 regions) revealed that the MSG+HFF diet can reshape composition of gut microbiota. Hamsters fed on this diet had much lower abundance of Bacteroidetes, but increased Euryarchaeota at phylum, class, order, family and genus levels relative to animals fed either diet alone. This finding was supported by the high ratio of Firmicutes/ Bacteroidetes in the MSG+HFF group, in agreement with findings of Feng *et al*. [53]. Alpha diversity and weighted Unifrac distance cluster analysis at the phylum level distinguished bacterial populations in animals fed with the combined diet from those given a single diet. In hamsters treated with MSG alone, changes in the gut microbiota were relatively slight, agreeing with a previous report in humans [18]. MSG led to increases in *Roseburia* (MN326554), *Citrobacter* (MN326558) and *Ruminococcus*_1 (MN326532). Increase of *Roseburia* after MSG consumption has been reported previously [18]. *Roseburia* is a taxon of anaerobic gut bacteria associated with butyrate production and negatively related to CKD progression [19, 55]. However, the increment of *Roseburia* (MN326554) level in this study might be due to MSG intake because glutamate in MSG can convert to butyrate [19, 54, 55]. The genus *Citrobacter* (Family Enterobacteriaceae) can produce the enzyme tyrosine phenol-lyase for converting L-tyrosine to 4-hydroxyphenylpyruvate and 4-hydroxyphenylpyruvate be further metabolized into *p*-cresol sulfate in the liver [56, 57]. In hamsters fed the HFF diet, an increase of *Dorea*, *Ruminococcaceae*_NK4A214_group (MN326535), dgA-11_gut_group (MN326549), *Desulfovibrio* (MN326533), *Bacteroides* (MN326550) and *Oligella* (MN326548) was found, similar to previous studies [58, 59]. *Desulfovibrio* (Family Desulfovibrionaceae) produces urea. An increment of urea level indicates kidney damage [54, 60]. Elevation of *Desulfovibrio* may thus serve as a microbiomarker for kidney injury. However, in relation to the MSG+HFF diet, a previous study in growing pigs found that dietary supplementation with fat and MSG increases the *Clostridium coccoides* group, *Fusobacterium prausnitzii*, *Peptostreptococcus productus*, *Faecalibacterium prausnitzii*, *Prevotella* and *Roseburia* in the cecum, but decreases *Bacteroides thetaiotaomicron* and the *Clostridium leptum* subgroup [53]. These differences might be explained by the different animal models used [61] and different diet compositions [62].

Among the changes in gut microbiota, we found an increase of *Methanobrevibacter* (MN326530) in the HFF+MSG group. This genus is an archaeon in the phylum Euryarchaeota. An increase in abundance of *Methanobrevibacter* may be associated with obesity [63] and kidney injury [64]. *Methanobrevibacter* promotes fermentation of polysaccharides by removal of H_2_, resulting in an increase in levels of short-chain fatty acids in the colon and host adiposity [54]. Elevated representation of *Methanobrevibacter* is also found in rats fed with a high-fat diet [65]. In addition, levels of beneficial bacterial genera such as *Lactobacillus* (MN326537) and *Allobaculum* (MN326542) [66] were lower in hamsters exposed to either MSG or HFF diets. *Lactobacillus* can be an antioxidant and reduce uremic toxins in serum [67, 68]. A decrease in *Lactobacillus* abundance might affect the level of uremic toxin. The lowest proportion of these beneficial bacteria was found in hamsters fed on the MSG+HFF diet, consistent with results from previous reports [20, 69]. In contrast, relative abundance of pathogenic bacteria, such as members of the *Escherichia-Shigella* (MN326536) genus, increased in hamsters given the MSG and/or the HFF diet. The highest abundance of this genus was seen in the MSG+HFF group, similar to the result from a previous report [70]. Taken together, the bacterial composition, Firmicutes/ Bacteroidetes ratio, as well as alpha and beta diversity indicate that the MSG+HFF diet increases gut dysbiosis in hamsters more than does either diet alone.

Gut dysbiosis causes alteration of gut-derived metabolites including TMAO, indoxyl sulfate and *p*-cresol sulfate, which are uremic toxins that contribute to kidney injury [45, 52, 71–74]. We found that a combination of MSG and HFF led to increased *p*-cresol sulfate level but decreased TMAO and indoxyl sulfate levels in urine compared to normal controls. *P*-cresol sulfate, a major component of urinary myelin basic protein-like material, causes renal tubular cell damage by inducing oxidative stress [74]. As noted above, we found that the highest relative abundance of *Akkermansia* (MN326539) and an increase in *p*-cresol sulfate levels were seen in the MSG+HFF group. The association between *Akkermansia* and *p*-cresol sulfate metabolite has been recently reviewed [75].

TMAO is another important gut-derived metabolite contributing to kidney injury by promoting renal functional impairment and regulating the profibrotic transforming growth factor-β(TGF-beta)/Smad3 signaling pathway [73]. Hamsters fed with a combination of MSG and the HFF diet had decreased TMAO levels in urine, but an increase of *Methanobrevibacter* (MN326530) in the feces. An association between *Methanobrevibacter* and a reduction of TMAO plasma metabolite has been reported previously [76]. Possibly, the reduction of TMAO level that was observed in urine might be due to activities of other gut microbiota. In contrast, mice fed with a high-fat diet for 16 weeks to induce obesity exhibited elevated circulating levels of TMAO, resulting in renal interstitial fibrosis and dysfunction [73]. The different findings suggest that ingredients of diets, duration of exposure and species of animal used can all affect gut dysbiosis and host-microbial co-metabolism. Taken together, these changes could contribute to cause kidney injury [46, 52, 73]

## Conclusions

A combination of MSG and HFF diets increased severity of kidney injury and altered gut microbiota and urine metabolites much more than did either diet alone. Our findings should raise awareness about the important role of MSG and HFF diets in development of human kidney disease and might indicate intervention strategies that could be used to limit kidney disease.

## Supporting information

S1 Fig Representative of ^1^H nuclear magnetic resonance (NMR) spectra of urine samples obtained from a normal control (A), monosodium glutamate-treated (MSG) individual (B), high-fat and high-fructose diet-treated (HFF) individual (C); and a hamster given the MSG and HFF diet (MSG+HFF) (D). 3-IS = 3-indoxyl sulfate; P-CS = *p*-cresol sulfate; TMAO = trimethylamine *N*-oxide.
(TIF)
