# Supplementary figures and images for: A combination of monosodium glutamate and high-fat and high-fructose diets increases the risk of kidney injury, gut dysbiosis and host-microbial co-metabolism
Source: PLoS One. 2020 Apr 8;15(4):e0231237. doi: 10.1371/journal.pone.0231237 (PMC7141667; doi:10.1371/journal.pone.0231237)

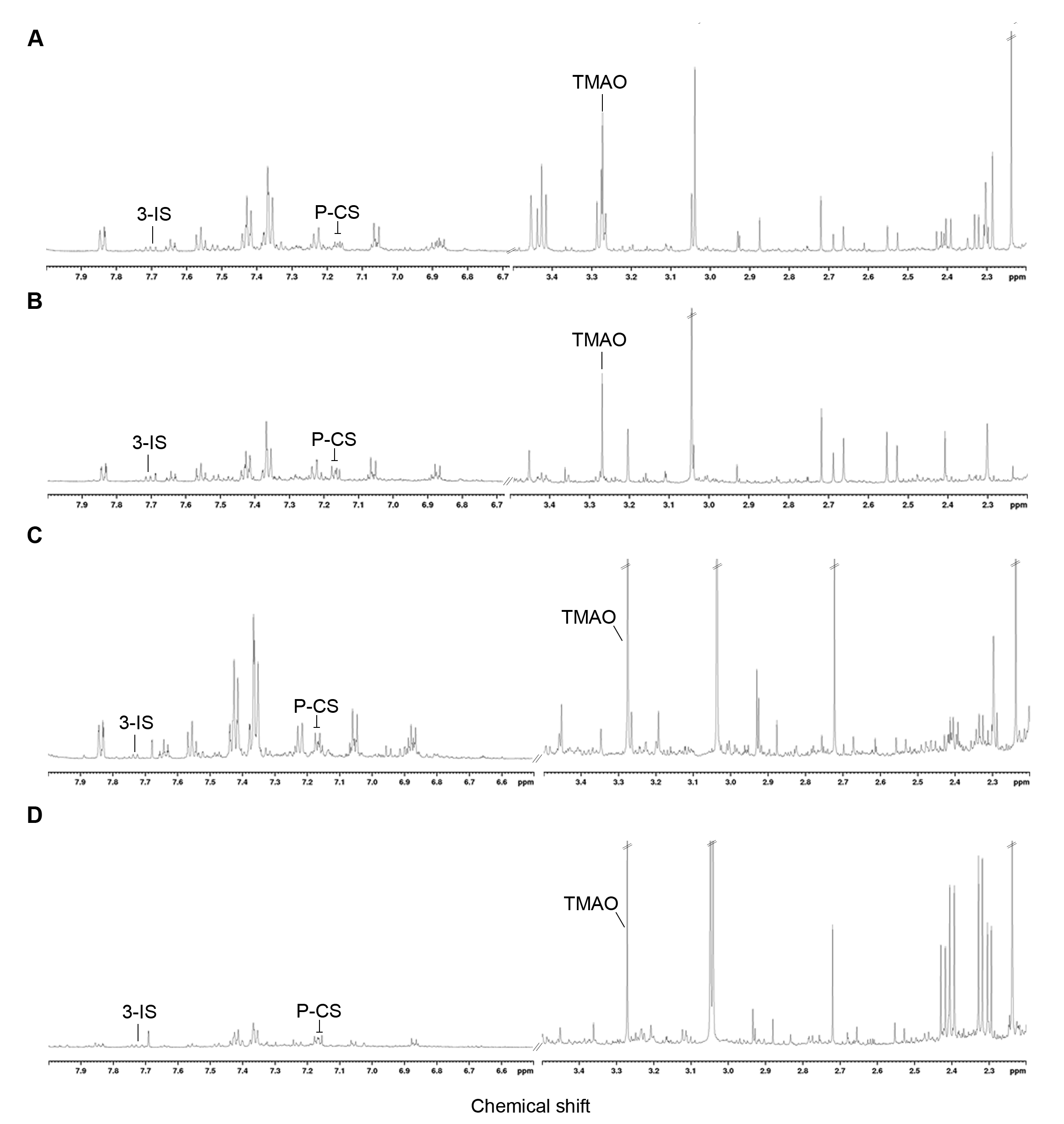

Supplement: S1 Fig — Representative of 1H nuclear magnetic resonance (NMR) spectra of urine samples obtained from a normal control (A), monosodium glutamate-treated (MSG) individual (B), high-fat and high-fructose diet-treated (HFF) individual (C); and a hamster given the MSG and HFF diet (MSG+HFF) (D). 3-IS = 3-indoxyl sulfate; P-CS = p-cresol sulfate; TMAO = trimethylamine N-oxide. (TIF) [file pone.0231237.s001.tif]
